# Scaffolding for assessment success: using gradual release of responsibility to support resident transition to competency-based medical education

**Published:** 2019-07-24

**Authors:** Rebecca Pero, Laura Marcotte

**Affiliations:** 1 Queen’s University, Ontario, Canada

## Abstract

**Implication Statement**

In competency-based medical education (CBME), assessment is learner-driven; learners may fail to progress if assessments are not completed. The General Internal Medicine (GIM) program at Queen’s University uses an educational technique known as scaffolding in its assessment strategy. The program applies this technique to coordinate early assessments with specific scheduled learning experiences and gradually releases the responsibility for assessment initiation to residents. Although outcomes of this innovation are still under investigation, we feel it has been valuable in supporting resident assessment capture and timely progression through stages of training. Other residency training programs could easily implement this technique to support the transition to Competency by Design.

## Introduction

In competency-based medical education (CBME), residents must demonstrate competence in specialty- specific clinical tasks.^1,2^ Assessment in CBME is learner-driven; residents must understand their learning objectives, seek out assessment opportunities and identify gaps in their assessment portfolio.^3^ However, as residents adjust to a new learning and working environment, operational aspects of CBME (i.e., what assessments to trigger and when to do so) may present an additional cognitive challenge.^4^ This adjustment period may result in a lack of assessment data, which may limit resident learning and possible advancement within the program.

## What is Scaffolding?

Scaffolding is a temporary support that helps a learner accomplish a task they may not be able to complete independently.^5^ With an aim to support resident learning and progress, as well as programmatic decision-making, we applied scaffolding through the instructional practice known as Gradual Release of Responsibility (GRR). This follows an “I do, we do, you do” series of three steps.^6^ In the two-year subspecialty program in General Internal Medicine (GIM) at Queen’s University, we introduced a scaffolding technique as part of our assessment strategy at the outset of the move to CBME to ensure adequate assessment of all the program’s entrustable professional activities (EPAs) (see Table 1). An entrustable professional activity is a “task in the clinical setting that may be delegated to a resident by their supervisor once sufficient competence has been demonstrated.”^7^ Initially, our program provided distinct moments where assessment information was captured (i.e., the program’s version of “I do”). In subsequent stages of training, there was a mix of program- and resident- initiated assessments (i.e., “we do”), and later, residents recognized assessment opportunities and captured their assessment data independently (i.e., “you do”) (see Table 1). Reporting the implementation of a CBME assessment program did not require a submission to the Queen’s University Ethics Review Board as per the Tri-Council Policy Statement 2, article 2.5.

**Table 1:** Assessment capture by EPA – sample of GIM EPAs to illustrate scaffolding strategy *

*Stage of Training / EPA*	*When / Where*	Who**
**Transition to Discipline**		
**D1** – history & physical exam	First GIM fellows’ clinic	program
**D2** – management of an acutely unstable patient	PGY-4 academic half day (simulation lab) x 4	program
**Foundations of Discipline**		
**F1** – GIM assessment of common patient presentation	GIM Fellows’ clinic / other clinical contexts	program / resident
**F2** – ongoing management of common GIM presentations	Relevant GIM inpatient rotations, weekly	program
**F3** – GIM approach to complexity / ambiguity	GIM Fellows’ clinic / other clinical contexts	program / resident
**Core of Discipline**		
**C1** – Assessing and managing complex inpatients	Any clinical inpatient context	resident
**C2** – Assessing and managing complex outpatients	GIM Fellows’ clinic	resident
**C3** – Peri-operative management rotations	Each GIM Pre-op Clinic, can also be assessed on call, on consult	program / resident
**C7** – End of life / supportive care	any clinical context	resident
**C8** – Critical care	- weekly assessment on ICU rotation - Resuscitation assessment at any time, any clinical context	program / resident
**Transition to Practice**		
**P1** – Manage the system	any clinical context	resident
**P2** – Complex communication	any clinical context	resident

* Note that these EPAs are Queen’s specific EPAs predating GIM’s official Royal College CBD process. They are similar to but not exactly the same as the ultimate GIM CBD EPAs being implemented in July 2019.

** Faculty may also initiate assessments at any time.

## Practical application of gradual release of responsibility in General Internal Medicine

During the Transition to Discipline stage of training (i.e., first month), our program oriented residents and assessed them on their basic internal medicine skills. We scheduled assessments during resident clinics and academic half days. The program “does” the assessment work, and the residents simply show up and demonstrate their knowledge and skills to complete the required number of assessments.

In the next stage, Foundations of Discipline (4-6 months), a structured assessment plan ensured some level of assessment completion while encouraging the residents’ developing understanding of the CBME paradigm, the assessment process, and their role within it. For one EPA, we sent weekly assessments to the appropriate clinical supervisor on all relevant GIM inpatient rotations. Assessments for two other EPAs were completed routinely in the GIM resident clinics, but were also completed in many other clinical contexts. We encouraged residents to independently initiate these same types of assessments in other settings (i.e., on non-GIM rotations). Thus, residents began to take charge of collecting the assessment information in a collaborative “we do” interaction.

In the Core of Discipline (12-16 months) and Transition to Practice (final 4-6 months) stages of training, residents need to be forward-thinking and conscious about when and where an EPA can be assessed (i.e., the “you do” step). The first two stages, having prepared residents for the added administrative load, lead to the scaffold being gently removed. Since the initial implementation of CBME in July 2017, nine residents in two cohorts have participated in our assessment strategy. The number of assessments required for each resident will depend on the resident’s performance and other factors as determined by the Competence Committee.

## Conclusion

To enhance resident learning and enable the capture of assessment data, the General Internal Medicine program at Queen’s University scaffolds its program of CBME assessment. Although we are still investigating the outcomes of this innovation, residents are progressing through the stages of training as expected, and residents in later stages of training are independently initiating a sufficient number of their own assessments for EPA achievement. This suggests that scaffolding may be an effective strategy to support resident success in CBME. We are in the midst of conducting a qualitative research study to understand whether residents perceive our assessment innovation as supportive to their success in the program. Other markers of success may include assessment data retrieved from the education technology system (i.e., assessments triggered by residents over time and per stage). We believe that this approach may be successfully applied in other residency training programs embarking on Competence by Design (CBD).

## References

[1] Competency-based medical education aligns with the Royal College of Physicians and Surgeons of Canada’s Competency by Design (CBD) framework.

[2] Competency-based education is “an approach to curriculum design and trainee assessment whose fundamental aim is to improve the training of health care professionals so that they deliver consistent, high-quality patient care” (Harris P, Bhanji F, Topps M, et al. Evolving concepts of assessment in a competency-based world. Medical Teacher. 2017;39:603-8). 28598736 10.1080/0142159X.2017.1315071

[3] A “robust and multifaceted” assessment system, that includes frequent, low stakes assessments, is a key component of CBME (Holmboe ES, Sherbino J, Long DM, et al The role of assessment in competency-based medical education. Medical Teacher. 2010;32:676-82). 20662580 10.3109/0142159X.2010.500704

[4] Sweller J Cognitive load during problem solving: Effects on learning. Cognitive Science. 1988;12:257-85.

[5] Fry H, Ketteridge S and Marshall S Understanding Student Learning. In: Fry H, Ketteridge S and Marshall S, eds. A Handbook for Teaching and Learning in Higher Education: Enhancing Academic Practice, Third Edition. New York, NY: Routledge 2009:8-26.

[6] Fisher D and Frey N Better Learning Through Structured Teaching: A Framework for the Gradual Release of Responsibility, Second Edition. Alexandria, VA: ASCD 2013.

[7] Royal College of Physicians and Surgeons of Canada 2019 [Internet]. Available at: http://www.royalcollege.ca/rcsite/canmeds/about/faq-canmeds-e. [Accessed: March 26, 2019].

